# Comparative in-vitro microscopic evaluation of vertical marginal discrepancy, microhardness, and surface roughness of nickel–chromium in new and recast alloy

**DOI:** 10.1038/s41598-023-40377-1

**Published:** 2023-10-04

**Authors:** Gotam Das, Saurabh chaturvedi, Talib Amin Naqash, Muhammad Waqar Hussain, Shahabe Saquib, Ghazala Suleman, Abdulelah Sameer Sindi, Shabina Shafi, Rania A. Sharif

**Affiliations:** 1 Department of Prosthodontics, College of Dentistry, King Khalid University, 61421 Abha, Saudi Arabia; 2 Department of Prosthodontics, Bakhtawar Amin Medical and Dental College, Multan, Pakistan; 3 Department of periodontics, Datta Maghe Institute of Higher Education & Research, Deemed to be University, Warda, 442001 India; 4 Department of Restorative Dental Sciences, College of Dentistry, King Khalid University, Abha, Saudi Arabia; 5 Specialist Pediatric Dentist, Saudi Dent Group Khamis Mushayt, Mushait, Saudi Arabia

**Keywords:** Structural materials, Dentistry

## Abstract

Reusing of alloy has become a need of time due to the increasing demand, depletion of resources, and substantial increase in their price. The alloys used require a long-term stay in the oral cavity exposed to a wet environment, so they must have good wear resistance, biocompatibility, and mechanically good strength. In this study, the vertical marginal discrepancy, surface roughness, and microhardness of the new and recast nickel–chromium (base metal) alloys were evaluated. 125 wax patterns were fabricated from a customized stainless steel master die with a heavy chamfer cervical margin divided into 5 groups. Each group had 25 samples. Group A: 25 wax patterns were cast using 100% by weight of new alloy, Group B: the casting was done by using 75% new alloy and 25% alloy by weight, Group C: wax patterns were cast using 50% new alloy and 50% alloy, Group D: 25% new alloy and 75% alloy and Group E: 100% recast alloy. The vertical marginal discrepancy was measured by an analytical scanning microscope, microhardness was tested on a universal testing machine, and surface roughness was on a tester of surface roughness. Castings produced using new alloys were better than those obtained with reused alloys. Alloys can be reused till 50% by weight along with the new alloy and accelerated casting technique can be used to save the lab time to fabricate castings with acceptable vertical marginal discrepancy, microhardness, and surface roughness. This indicated that 50% recasting of (Ni–Cr) can be used as a good alternative for the new alloy from an economical point of view.

## Introduction

High-noble and predominantly base metal alloys (such as Ni–Cr) have been used to make cast dental fixed prostheses. High-noble alloys were utilized due to their biocompatibility and corrosion resistance^1^. However, due to their ease of availability and low cost, Ni–Cr alloys became widely used. Ni–Cr alloys have good physical properties, including increased hardness, high tensile strength, and low density. Compared to other materials, the adoption of Ni–Cr alloys is enhanced by their lower weight and typically more acceptable mechanical qualities than high-noble alloys^2^.

In the present economy, dentists and technicians must be aware of the costs concerning the alloys they use for fixed dental prostheses (FDPs)^3,4^. To further reduce costs, previously used melted sprues and buttons of Ni–Cr may be mixed with new metal to fabricate restorations with minimal expenditure for dental laboratories. However, previously recast alloys contained different impurities from the castings^5^. These contaminations from old recasting methods can change the microstructure, chemical composition, cytotoxicity of alloys, grain dimensions, and corrosion^2,6^.

The most commonly used Ni-based alloys have 11 wt% and 25 wt% Cr. Recasting Co–Cr and Ni–Cr alloys also release Fe and Cu into cell-culture media. Adding at least 50% new alloy to recast old alloy for porcelain-fused-to-metal restorations does not significantly affect mechanical or physicochemical properties^7^. Additionally, the physical and chemical properties of Ni–Cr alloys do not undergo major changes after numerous recastings, and there is no direct association between the physicochemical characterization of Ni–Cr alloys with dissimilar chemical compositions and in vitro biological assessment tests. Different researchers have continued to study the ratios of fresh to recast alloys needed to obtain an alloy with satisfactory clinical performance^3,4^.

Several researchers have assessed the effect of recast Ni–Cr alloys on the marginal precision of crowns and found that maximal marginal accuracy is achieved in 100% new alloy castings, while minimal fitness is observed in 100% recast alloy castings. However, the marginal fit with 50% new and 50% recast alloy was still clinically satisfactory^1^. According to research the factors contributing to faulty castings include a decrease in microhardness and an increase in surface roughness. The exterior of the casting requires extra effort in finishing and polishing, while the tissue surface may inhibit the appropriate seating of the casting. These damaged areas can alter test results and affect physical characteristics^8^.

Reusing the base metal alloy can decrease predictable costs by 30–40%. Recycling alloys is highly favorable from both environmental and economic perspectives, as it reduces the number of natural resources used and contamination caused by casting fumes and mineral extraction. However, the impact of this process on the quality of dental prostheses is not yet fully understood and requires further research^9,10^.

The surface roughness and microharness of two different base metal alloys subjected to different casting techniques was examined, as well as the influence of surface roughness on surface loss after polishing, and it was concluded that decreased surface roughness was observed in base metal alloys fabricated by vacuum casting as opposed to base metal alloys fabricated by acetylene-oxygen flame casting. For all studied specimens, there were no significant variations in mass loss following polishing^11^.

Metal-ceramic alloy frameworks produced utilizing a CAD-CAM workflow exhibited much lower marginal discrepancies than those produced using a typical Ni–Cr approach, with the milled group having the greatest marginal match among the three test groups. Manual refinement improved the marginal fit of all groups greatly^12,13^.

There are only a few studies on the recasting of Ni–Cr alloys in dental research, with some evaluating the characteristics of the Ni–Cr alloy by casting the used alloy, while others have analyzed by adding fresh content to the casted alloy^14^. It would be of great scientific benefit to study the characteristics of recast alloys in-depth and provide guidelines to prosthodontists and dental laboratory technicians^15,16^.

The present study aims to evaluate the vertical marginal discrepancies and mechanical properties, such as microhardness and surface roughness, of base metal (nickel–chromium) alloys at various proportions of new and recast alloys: 100% new alloy, 75% new and, 25% recast, 50% new and recast, 25% new and 75% recast, and 100% recast alloy. This study aims to investigate the feasibility of reusing buttons and sprues obtained from casting new alloys and to compare the differences in marginal precision, surface roughness, and microhardness between recast alloys and the control (new alloy). These combined methods will contribute to understanding the effects of recasting on the properties of Ni–Cr alloy castings.

## Materials and methods

A customized stainless steel master die with a base was created to replicate the prepared tooth. The die had a height of 9 mm from the occlusal surface to the finish line and had a taper of 6°. A chamfer finish line was machined into the die, situated 1 mm above the base. The diameter of the die near the base was 9 mm. An offset angle was added at the axio-occlusal line angle to ensure accurate reseating of the crowns.

Wax patterns were made by applying a thin layer of die lubricant on the master die and using inlay wax (HARVARD, Richer and, Hoffman Harvard Dental-Gmbh, Germany). After the wax had cooled, wax copings were removed from the master die. The margins were adjusted and refined using wax carving tools, and the wax patterns were divided into 5 groups, each containing 25 wax patterns. The wax patterns were then cast using different percentages of new (75% Ni, 15% Cr, 5% Mo, and 1.6% Be35, Thermabond alloy super cast, MFG, Los Angeles, CA, USA) and recast Ni–Cr alloy, and again divided into 5 groups, each containing 25 crowns.

Group A: 100% new alloy (control).

Group B: 75% new alloy and 25% recast alloy.

Group C: 50% new alloy and 50% recast alloy.

Group D: 25% new alloy and 75% recast alloy.

Group E: 100% recast alloy.

After the wax elimination process, the investment was heated in a furnace (Ney Vulcan, Dentsply Ceramco, York, Pennsylvania, USA) until all the wax had vaporized. The castings were then made using an induction-casting machine with different percentages of new and recast Ni–Cr alloy. Once the castings were recovered, they were mechanically divested of the investment and cleaned to remove any residual investment material. This effort was done to investigate the phenomena of fusion in Ni–Cr alloys and determine the most acceptable composition with melting temperatures ranges 1100–1200 °C; to make the alloy simple for casting and fabrication for dental prosthesis.

Each alloy crown was then placed on the master die corresponding to the prepared tooth, and a force of 7.4 pound-force was applied in a vertical direction for one minute. The vertical marginal discrepancy was then determined by measuring the vertical space between the finish line of the test die and the margin of the alloy crown at four 90° sites using an analytical scanning microscope (JSM-6360LA, Joel Ltd., Japan). The sites were verified by arbitrary locations of the grid. Marginal gaps were measured to the nearest micron on each casting at the four predetermined sites on the base of the stainless steel die separated by 90°. The same procedure was followed to record the vertical marginal gap for each of the 10 test samples belonging to the two test groups. An accelerating voltage of 15 kV under × 200 magnification was used for the assessment of marginal disrecpancy. The measurements thus obtained were tabulated and statistically analyzed.

### Microhardness measurements

Microhardness Tester REMET HX-1000 was used for indention to measure the value of the Vickers microhardness. These tests were performed based on guidelines UNE-EN ISO 6507-1:2006, load allocation of 100 g during 15 s. every sample got a minimum of 5 indentations were made and the mean was measured so it is called Vickers hardness (HV).

### Surface roughness

The surface roughness was calculated by the help of a profilometer (SurfCorder SE 1700; Kosaka, Tokyo Japan) with a diamond stylus tip for cylindrical surfaces (AG5; Kosaka), that touched the surface at a steady speed of 0.05 mm/s with the force of 0.7 mN. It is described by the height parameter, Ra (in micrometers, mm), which is the arithmetical mean of the fixed rates of the profile within the length assessed. To characterize surface roughness the cut-off value was set at 0.08 mm. Statistical evaluation of surface roughness was carried out using a standard of 3 values of surface roughness parallel to the long axis at the central segment of each sample.

### Statistical analysis

Results were designed with a mean and standard deviation. The variance was analyzed using ANOVA. The difference between groups was identified by post hoc Turkey’s test.

## Results

**Figure 1:**
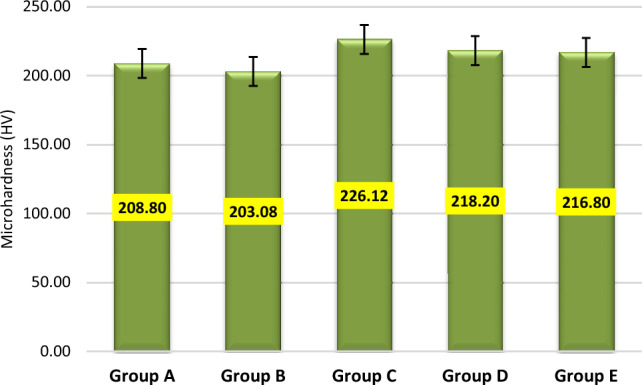
Intergroup comparison of microhardness.

**Figure 2:**
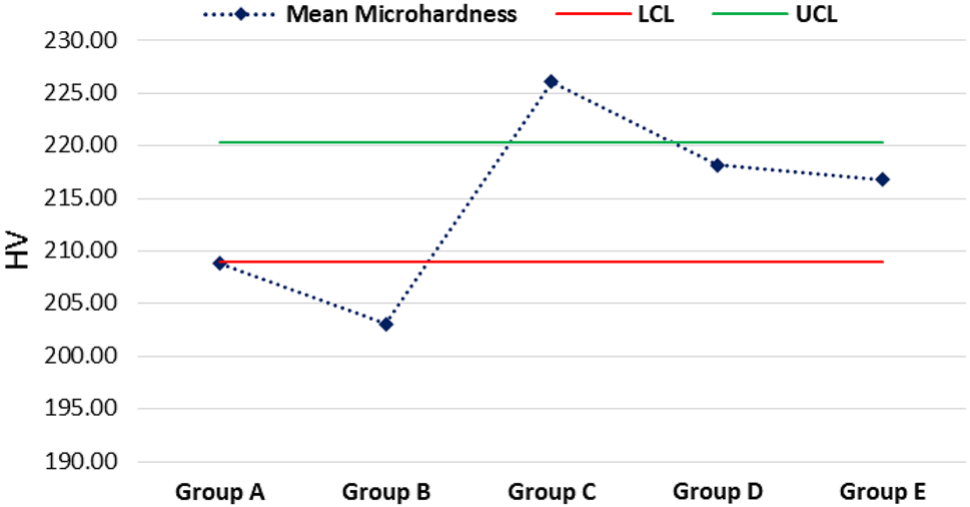
Quality control analysis of microhardness.

**Figure 3:**
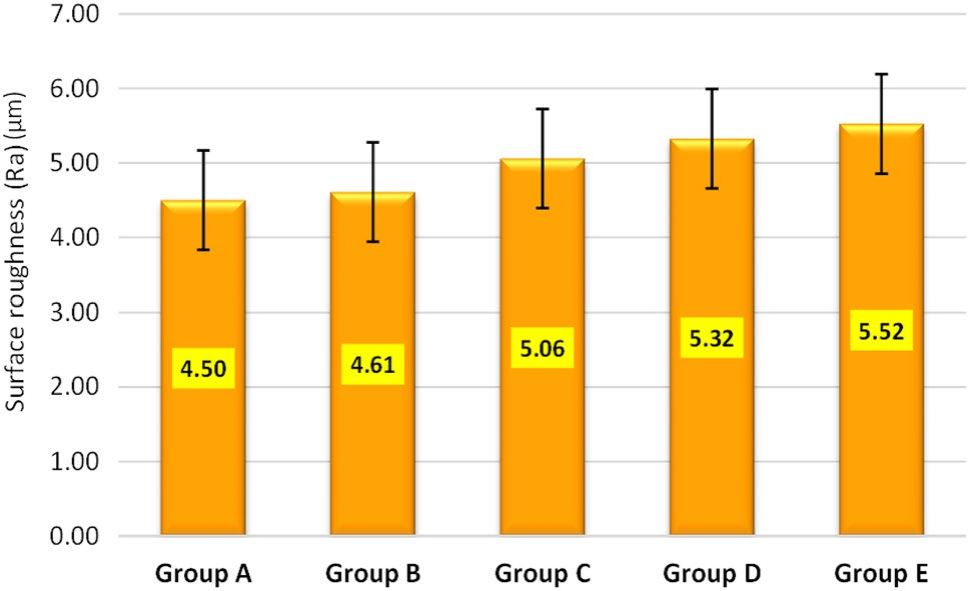
Intergroup comparison of surface roughness.

**Figure 4:**
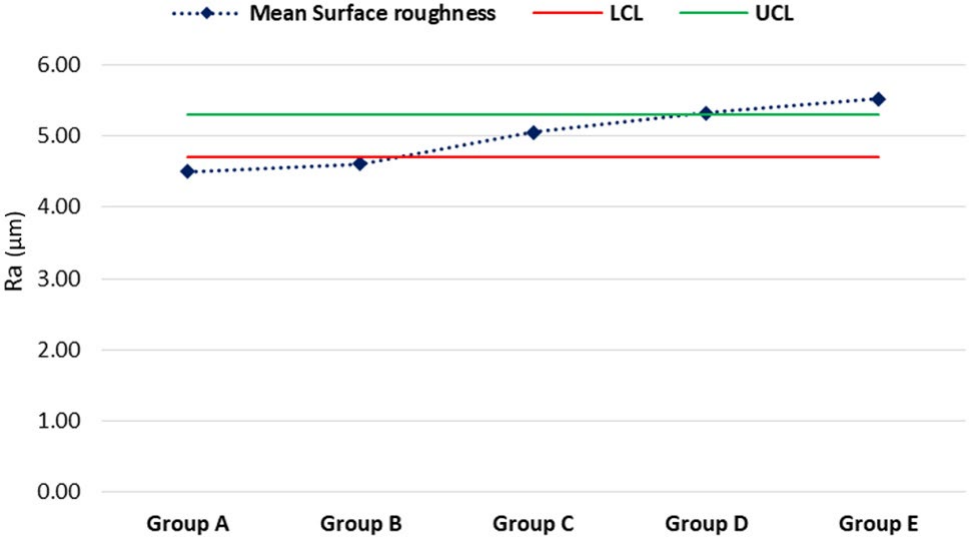
Quality control analysis of surface roughness.

**Figure 5:**
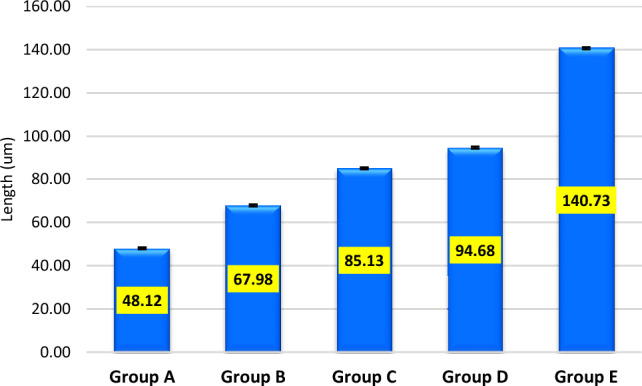
Intergroup comparison of length.

**Figure 6:**
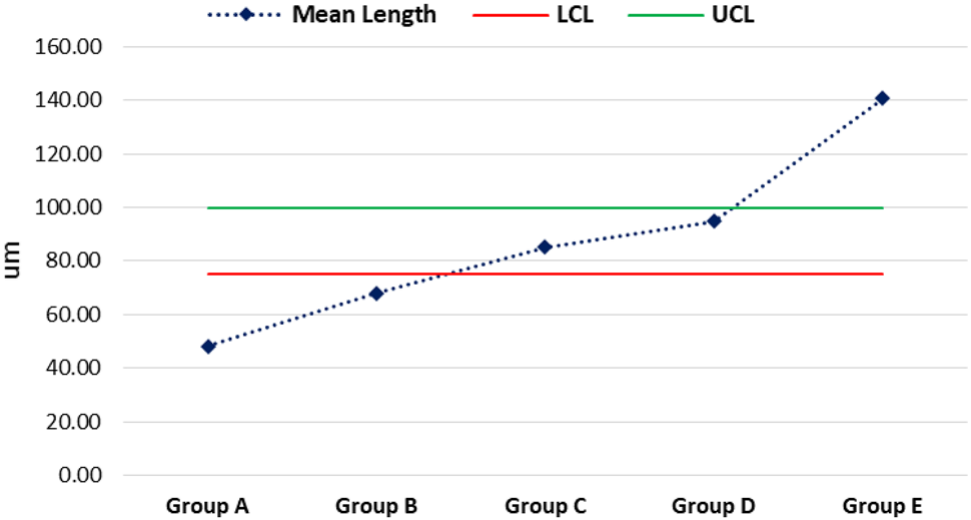
Quality control analysis vertical length.

**Figure 7:**
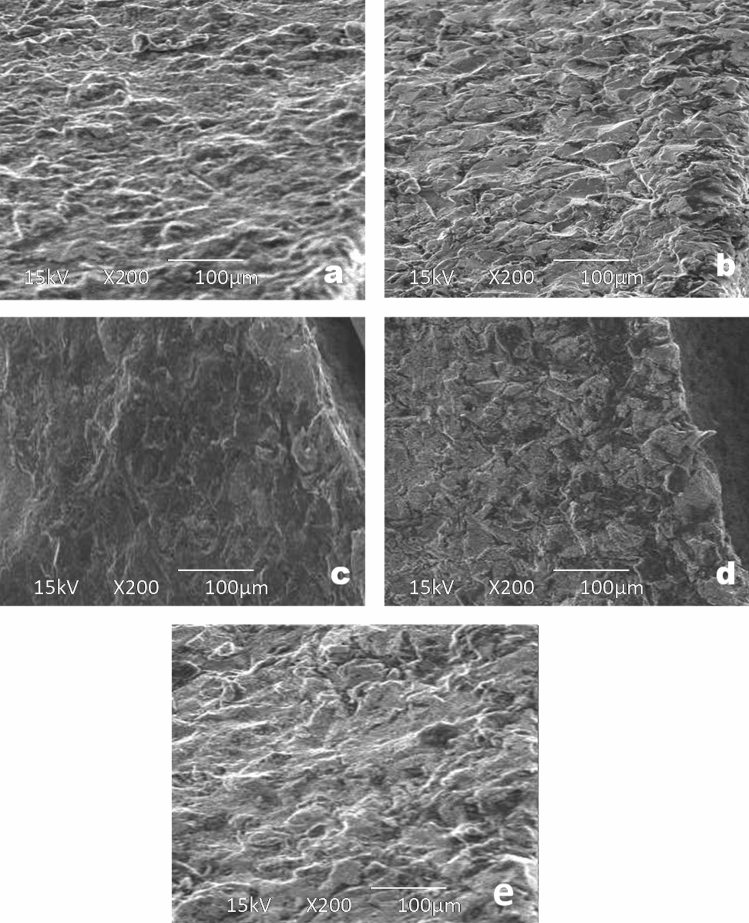
Surface roughness in subgroups from (**a**) to (**e**).

**Figure 8:**
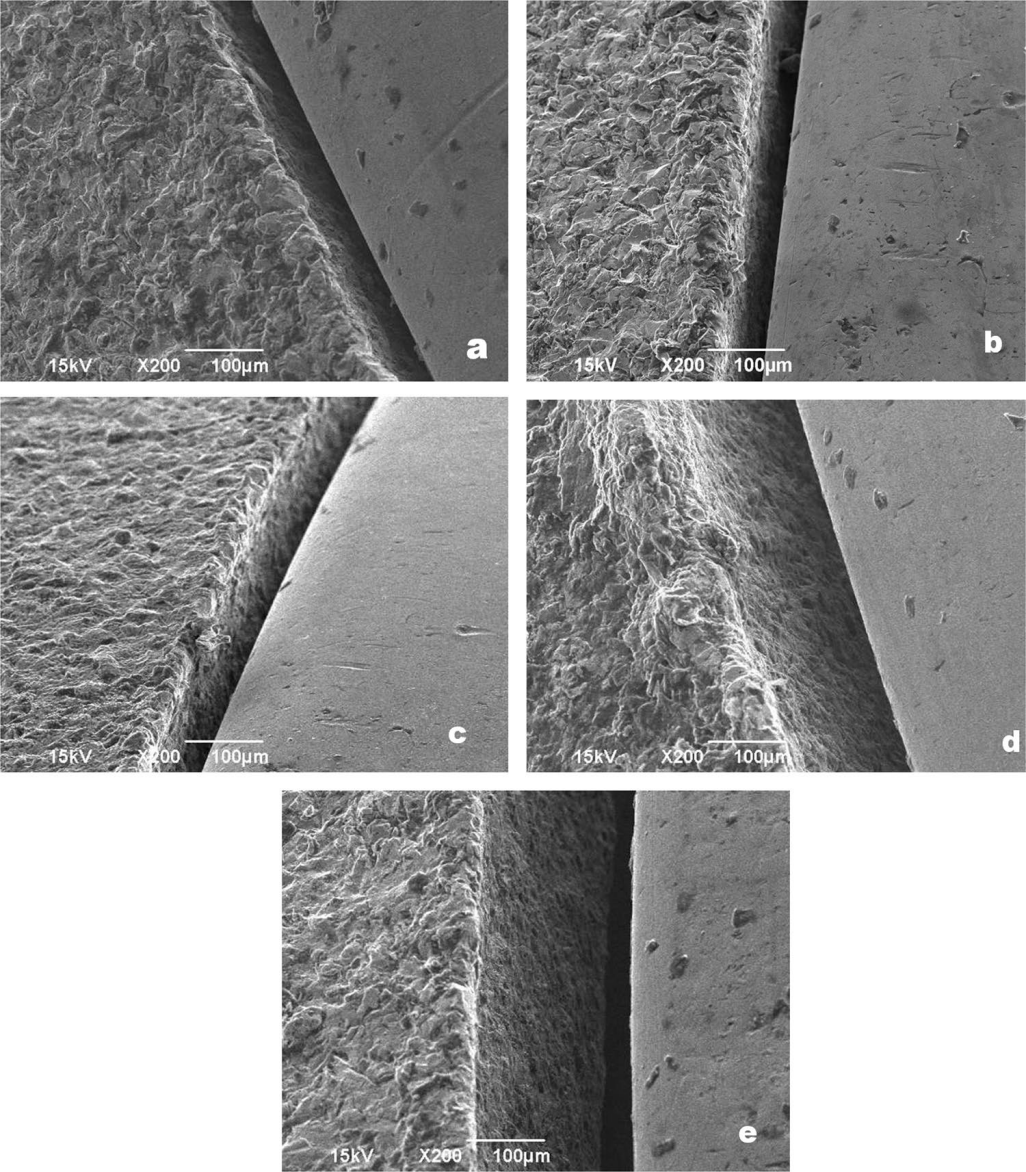
Marginal discrepancy in all subgroups from (**a**) to (**e**).

The mean maximum microhardness was 226.12 ± 9.98 HV found for group C while the minimum was 203.08 ± 17.97 HV found for group B. A significant difference was observed in mean microhardness among the groups (p < 0.001) as shown in Fig. 1. According to quality control analysis, the mean microhardness was significantly high for group C as the corresponding point on the control chart was lying above the UCL (upper 95% confidence line), while the mean microhardness was significantly low for group A & group B as the corresponding points on control chart were lying below the LCL (lower 95% confidence line) as in Fig. 2. In Fig. 3, the mean maximum surface roughness was 5.52 ± 0.719 Ra found for group E while the minimum was 4.50 ± 0.674 HV found for group A. A significant difference was observed in mean surface roughness among the groups (p < 0.001). According to quality the control analysis, the mean surface roughness was significantly high for group E and group D as the corresponding points on control chart were lying above the UCL (upper 95% confidence line), while the mean surface roughness was significantly low for group A & group B as the corresponding points on control chart were lying below the LCL (lower 95% confidence line) shown in Fig. 4. In Fig. 5, the mean maximum length was 140.73 ± 1.605 µm found for group E while the minimum was 48.12 ± 0.474 µm found for group A. A significant difference was observed in mean vertical length among the groups (p < 0.001). It has been shown that the vertical length in all groups as the ratio of the reused alloy increased the vertical discrepancy also increased. According to quality control analysis, the mean length was significantly high for group E as the corresponding point on the control chart was lying above the UCL, while the mean length was significantly low for group A and group B as the corresponding points on the control chart were lying below the LCL as shown in Fig. 6. It has been shown that the surface roughness in all groups as the ratio of the reused alloy increased the surface roughness is also increased as shown in Fig. 7. The marginal discrepancy is shown in Fig. 8 as in alloy with less reuse alloy the gap is less but as we increase the percentage of reuse alloy the gap is increased significantly (Supplementary Information 1).

## Discussion

The effect of recasting of Ni–Cr on fit of cast restorations have been investigated in experimental conditions through measuring the marginal discrepancy, surface roughness and microhardness in new and reused castings. The results of the study showed that increasing the percentage of reused Ni–Cr for casting increased the marginal gap discrepancy, surface roughness and microhardness. However, statistical analyses revealed that these differences were not significant. These findings agreed with the results of^17^ for combining new and recast alloys. In addition, the lowest valued recorded for the all three factors was in reused alloy may be explained by the potential loss of trace metal (e.g., Fe) present in the as received alloy during the remelting and re-casting procedure through volatilization or oxidation resulting in excessive solidification contraction^2,18^.

This study also demonstrated better marginal discrepancy of new Ni–Cr alloy than reported for reused alloy. Palaskar et al.^3,4^ studied the discrepancy of new and recast alloy before and after addition of used alloy. They concluded that the marginal discrepancy of new alloy crowns was significantly better than that of recast alloy. Atluri et al.^19^ reported the discrepancy of recast alloy made by 100% reused alloy was greater than that reported in the present study for new alloy. The better fit of new alloy can be explained by the improved castability which could be attributed to differences in melting temperature, thermal expansion coefficient, and density. It is important to note that the recasting of base metallic alloys can alter the basic structure, and mixing new and old metal can affect direct marginal differences, surface roughness, and microhardness of base metal alloys, leading to changes in the concentration of major elements such as Ni, Cr, Mo, and other minor elements including Cu, Zn, and Ag^1,20^.

In the current study, the changes in marginal discrepancy were as small as 48.12 ± 0.474 μm for the new mixture compared to the recast mixture with different percentages: 25% old (67.9822 ± 0.965 μm), 50% old (226.12 ± 2.213 μm), and 94.60% ± 7 μm old (140.72 ± 1.605 μm). Wagner et al.^21^ calculated the physical characteristics of base metal alloys and suggested that they can be used for four generations without significant variation. Nandishet al.^22^ confirmed that there was no significant deterioration in Ni–Cr alloy recasting for up to 10 generations, and that mixing used metal with new metal and recasting 100 times did not create significant deteriorating changes in the base materials, microstructure, or clinical quality. The authors stressed the importance of adhering to uncontaminated materials to reduce pollution and negative impacts on the material. Wagner et al.^21^ confirmed that easily oxidized compounds are dispersed without throwing and mixing with oxygen because the metal near the surface changes in alloy formation. The changes described in the statement may occur concurrently during the second stage and may affect the disintegration of the mixture. As a result, the high concentration of the mixture may lead to the formation of oxidation by-products^23^.

Several studies have been conducted by various authors on the impact of reusing base metal alloys on the marginal accuracy of crowns. These studies have shown that the maximum marginal fitness is achieved with 100% new alloy castings; while the minimum fitness is achieved with 100% reused alloy castings. However, the marginal fit with a combination of 50% new and 50% reused alloy was found to be slightly less than that of 100% new alloy, but it was still considered clinically acceptable^19,20,24^.

The results of the current study are different from the study conducted by Khaledi et al.^23^. He found that the elemental structure of high gold alloy remained constant throughout recasting methods. However, according to him, the maximum number of castings failed in clinical situations due to corrosion or tarnish, while the nobility declined or the silver–copper ratio was changed.

In the present study, the suggested value for the vertical marginal discrepancy of the new alloy was 48.12 ± 0.474 μm, while the overall recast alloy resulted in a discrepancy of 226.12 ± 2.213 μm. This shows that the marginal accuracy was acceptable when 25% or 50% recast alloy was used. The findings of the present study are consistent with those of other studies that have reported that the addition of new and old base metal alters the compositional balance of a Ni–Cr base metal alloy, leading to minor changes in marginal accuracy^8,25^.

Bauer calculated the results of three different casting procedures on the marginal accuracy of a high noble alloy^25^. However, although statistically significant, the changes in vertical marginal discrepancy were not clinically significant, with a marginal accuracy of less than 25 μm for all casting conditions. In a recent study, the cervical and internal fit of complete metal crowns that were cast and recast with Pa–Ag alloy were evaluated for three different marginal configurations. The authors found that the as-received alloys showed significantly better marginal adaptation in new alloys than in recast alloys. Three studies, in which the authors measured the marginal gap using a stereomicroscope, were reviewed, but the results were not consistent. Bauer et al.^25^, Yun et al.^26^ and Gyamfi et al.^27^ concluded that recasting could be performed without major limitations.

The outer surface of all castings had different degrees of surface roughness, which affects the physical behavior of the casted metal. The present study showed that changes in surface roughness were minimal at 4.5 ± 0.67 μm for castings with the new alloy, while compared to the recast alloy with 25% old at 4.61 ± 0.67 μm, 50% old at 5.06 ± 0.57 μm, 75% old at 5.32 ± 0.70 μm, and 100% old at 5.52 ± 0.71 μm. Changes in surface roughness in new or recast were not significantly different. Thus, it may be hypothesized by this study that causes for surface roughness are likely associated with the casting alloy, either new or recast, and alterations that occur may be the loss of certain trace elements, including manganese, chromium, and molybdenum, oxide layer formation^2,9,13,28^ and incorporation of oxygen and nitrogen^21,29,30^.

Lopes et al.^31^ and Chao et al.^32^ reported that only minor changes occur in microhardness values on recasting. Bajoghli et al.^33^ and Walczak et al.^34,35^ confirmed that in each generation of recast, there is a 0.01% loss of components from the alloy, which may contribute to the difference in microhardness. In our study, the values of microhardness were slightly higher in the new Ni–Cr alloy group A than in the recast alloy group B. Therefore, recast alloy can be utilized in fixed prosthodontics with respect to microhardness. Usman et al.^36^ conducted similar research, concluding that the marginal fitness of the new alloy was superior to that of the recast alloy. Usman claimed that the marginal gap between recast and fresh alloys is caused by oxidization, vaporization, and porosity.

Finally, it could be summarized that recast Ni–Cr steel alloy confirmed correct results regarding measured microhardness belongings that might be used instead of as-received alloy that is extra pricey for fabrication of constant prosthesis while recasting in guided moderate decrease the microhardness of Ni–Cr-alloy in distinctive ranges for distinctive ratios of recasting alloy so it's far most well known may be to use the recast alloys for the fabrication of dental prosthesis.

The study by Ramírez et al.^37^ suggests that the scrap generated in dental laboratories can be recycled by melting it under controlled conditions. On the other hand, the study by Walczak et al.^34,35^ found that the release of constituents increased significantly with the increase in the percentage of recast material used in casting procedures, which led to a rise in cytotoxicity.

In this study, the changes observed in vertical marginal discrepancy, surface roughness, and microhardness were generally minimal for all types of alloys, suggesting that recast alloys can be used in different percentages for fixed prosthodontics. A study by Walczak et al.^34,35^ also reported that at least 50% new Ni–Cr alloy can be incorporated in copings for porcelain to metal restorations. However, it is important to note that the quality of the restoration should not be compromised at the expense of the properties of the alloy. The small reduction in the concentration of Ni and Cr is unlikely to have any clinically significant impact on the physical characteristics or resistance to corrosion of the cast alloys^38^.

To promote the reuse of alloys, dental laboratories should establish effective methods for metal handling and accounting. Additionally, manufacturers could develop cost-effective methods for improving the cast alloy to encourage laboratories to return the large and cumbersome buttons that tend to accumulate.

## Conclusions

Based on the results obtained and the limitations of this study, the following conclusions can be drawn.

There was a minor difference in surface roughness and microhardness, but a significant difference in vertical marginal discrepancy among the groups cast using different combinations of Ni–Cr alloys. The addition of reused alloy up to 50% was found to be acceptable due to increased marginal discrepancy and surface roughness among Groups A and B. However, a significant statistical variation was observed among Group III, IV, and V, indicating a weakening of physical properties when the content of the reused alloy is 50% or more. The increase in marginal discrepancy in the reused alloy may be due to changes in physical properties.

### Supplementary Information

Supplementary Information 1.

Supplementary Information 2.

## Data Availability

The datasets used and/or analysed during the current study available from the corresponding author on reasonable request.
