# Supplementary material for: Comparative in-vitro microscopic evaluation of vertical marginal discrepancy, microhardness, and surface roughness of nickel–chromium in new and recast alloy
Source: Sci Rep. 2023 Oct 4;13:16673. doi: 10.1038/s41598-023-40377-1 (PMC10551011; doi:10.1038/s41598-023-40377-1)
Supplement: Supplementary file 2 — Supplementary Information 2. [file 41598_2023_40377_MOESM2_ESM.docx]

Specimen 1.1

Length 45.320484 µm

Length 49.256802 µm

Length 48.670020 µm

Length 51.101345 µm

Specimen 1.2

Length 50.220484 µm

Length 47.345480 µm

Length 49.456720 µm

Length 46.234545 µm

Specimen 1.3

Length 47.412584 µm

Length 44.156802 µm

Length 48.124020 µm

Length 49.258345 µm

Specimen 1.4

Length 50.478484 µm

Length 48.258980 µm

Length 46.456720 µm

Length 48.125545 µm

Specimen 1.5

Length 46.748484 µm

Length 48.256802 µm

Length 47.225020 µm

Length 50.369845 µm

Specimen 2.1

Length 53.880484 µm

Length 49.254802 µm

Length 55.470020 µm

Length 55.001345 µm

Specimen 2.2

Length 51.468254 µm

Length 52.759310 µm

Length 49.913420 µm

Length 49.990387 µm

Specimen 2.3

Length 52.220484 µm

Length 49.345802 µm

Length 54.254020 µm

Length 55.325645 µm

Specimen 2.4

Length 51.468254 µm

Length 53.759310 µm

Length 50.453560 µm

Length 50.560787 µm

Specimen 2.5

Length 50.859647 µm

Length 52.754587 µm

Length 49.458960 µm

Length 51.445787 µm

Specimen 3.1

Length 57.138441 µm

Length 55.027719 µm

Length 52.474305 µm

Length 53.185353 µm

Specimen 3.2

Length 60.847228 µm

Length 57.724749 µm

Length 58.581791 µm

Length 57.104702 µm

Specimen 3.3

Length 52.789441 µm

Length 49.487719 µm

Length 51.454305 µm

Length 48.758353 µm

Specimen 3.4

Length 58.785228 µm

Length 56.748549 µm

Length 55.256791 µm

Length 53.104458 µm

Specimen 3.5

Length 56.685898 µm

Length 54.744589 µm

Length 53.258911 µm

Length 50.458968 µm

Specimen 4.1

Length 58.560697 µm

Length 60.320361 µm

Length 65.682029 µm

Length 61.558408 µm

Specimen 4.2

Length 58.124233 µm

Length 53.132558 µm

Length 59.576051 µm

Length 59.096075 µm

Specimen 4.3

Length 54.450697 µm

Length 60.140361 µm

Length 63.452029 µm

Length 60.589408 µm

Specimen 4.4

Length 60.458233 µm

Length 53.122558 µm

Length 58.576051 µm

Length 51.584075 µm

Specimen 4.5

Length 59.778533 µm

Length 52.472558 µm

Length 57.458051 µm

Length 54.454875 µm

Specimen 5.1

Length 59.837628 µm

Length 58.774749 µm

Length 57.981791 µm

Length 55.704702 µm

Specimen 5.2

Length 61.587697 µm

Length 59.321361 µm

Length 60.324029 µm

Length 62.543408 µm

Specimen 5.3

Length 59.787628 µm

Length 60.785449 µm

Length 58.251791 µm

Length 62.589602 µm

Specimen 5.4

Length 61.577897 µm

Length 61.321361 µm

Length 65.458029 µm

Length 63.256988 µm

Specimen 5.15

Length 62.145828 µm

Length 61.725899 µm

Length 57.458771 µm

Length 64.145870 µm
